# Dopaminergic agonist pramipexole improves memory and increases IL-10 production in LPS-challenged rats

**DOI:** 10.22038/ijbms.2021.50439.11488

**Published:** 2021-05

**Authors:** Anita Mihaylova, Nina Doncheva, Hristina Zlatanova, Delian Delev, Mariya Ivanovska, Yvetta Koeva, Marianna Murdjeva, Ilia Kostadinov

**Affiliations:** 1 Department of Pharmacology and Drug Toxicology, Faculty of Pharmacy, Medical University of Plovdiv, Bulgaria; 2 Department of Pharmacology and Clinical Pharmacology, Medical Faculty, Medical University of Plovdiv, Bulgaria; 3 Department of Microbiology and Immunology, Research Institute (RIMU), Faculty of Pharmacy, Medical University of Plovdiv, Bulgaria; 4 Department of Anatomy, Histology and Embryology, Medical Faculty, Medical University of Plovdiv, Bulgaria

**Keywords:** Amyloid, Cytokines, Hippocampus, Lipopolysaccharide, Neuroinflammation, Pramipexole

## Abstract

**Objective(s):**

A variety of cytokines are involved in cognitive functioning. Balance restoration between protective and degenerative neuro-inflammation is of great interest in newer therapeutic approaches. In the current study, we investigated the effect of pramipexole (PMX) on memory functions, hippocampal amyloid deposition, serum cytokines, and brain-derived neurotrophic factor (BDNF) levels in lipopolysaccharide (LPS) challenged-rats.

**Materials and Methods:**

Male Wistar rats were divided into 5 groups (n=8): control (saline), lipoppolysacharide (LPS 250 mcg/kg bw), and experimental groups (LPS and PMX 0.5, 1, and 3 mg/kg bw). Learning and memory were assessed by the novel object recognition test (NORT), Y-maze, and step-through test. Immunological and histological assays were performed.

**Results:**

In memory tasks, LPS-challenged rats showed reduction in the observed parameters. In NORT, PMX 1 mg/kg increased recognition index compared with controls, whereas the other two doses increased this index only against the LPS-control. In Y-maze, all doses of PMX significantly had increased alternation when compared with LPS. In the step-through test, only the lowest dose of PMX extended the latency compared with LPS. Histological examination revealed that PMX at doses of 0.5 and 1 mg/kg reduced amyloid deposition in the hippocampus. Interleukin (IL)-10 serum levels were elevated by 1 mg/kg PMX. Tumor necrosis factor (TNF)-α and transforming growth factor (TGF)-β1 serum levels remained under the detectable minimum in all experimental groups. PMX at all doses significantly decreased BDNF serum concentration.

**Conclusion:**

In rats with LPS-induced neuro-inflammation PMX improved hippocampal-dependent memory and exerted immuno-modulatory effects by increasing IL-10.

## Introduction

Neuroinflammation and neurodegeneration are primary hallmarks in the pathogenesis of various neurological conditions. In relation to different clinical phenotypes, such as Alzheimer’s disease and Parkinson’s disease, existing evidence reveals an association between neurodegeneration and cognitive impairment (1). Substantive data show that neuronal proinflammatory mechanisms play an essential role in cognitive decline (2). Activation of microglia and astrocytes is implicated in the inflammatory reaction within the central nervous system (3). Stimulated microglia leads to brain injury caused by oxidative stress and production of proinflammatory cytokines (4). In physiological conditions, both pro-inflammatory (such as TNF-α) and anti-inflammatory (e.g., IL-10) mediators are involved in normal cognitive functioning (5). Nowadays, the restoration of the balance between the protective and degenerative neuro-inflammation is of great interest in newer therapeutic approaches (6).

TNF-α is recognized, to some extent, as responsible for the functional consequences following neuroinflammation (7). At normal levels, TNF-α has an essential role in brain synaptic plasticity, learning, memory, and normal behavior (8). However, microglial activation leads to increased TNF-α expression with subsequent generation of reactive toxic radicals (9). TGF-β1 is considered a regulator of cell growth suggesting its involvement in the modulation of neurodegenerative processes (10). A study revealed that TGF-β1 plays a role in learning and memory enhancement (11). IL-10 exerts anti-inflammatory properties and plays an essential role in resolving neuroinflammation (6). It stimulates the TGF-β1 synthesis by astrocytes, which in turn, reduces microglial activation (12). Different glial cells produce trophic factors responsible for the survival of the neurons. BDNF is implicated in hippocampal and cortical neuronal growth, neurogenesis, and synaptic plasticity. Experimental data showed that BDNF is of great significance for learning and memory processes (13). 

The bacterial lipopolysaccharide (LPS) is a potent microglial activator, widely used in experimental studies to mimic the inflammatory processes underlying neurodegenerative disorders. The systemic administration of LPS causes neuronal cell death and increases amyloid beta levels leading to cognitive decline (14).

Pramipexole (PMX) is a relatively new agonist of dopamine D_3_/D_2_ receptors. It is more selective for D_3_ than D_2_ and D_4_ subtypes (15). Preceding studies demonstrated that PMX protects neurons from reactive oxygen species in *in vitro* and *in vivo* conditions. Most likely its neuroprotective effects are due to immunomodulatory properties (16).

The objective of the current study was to investigate the effect of PMX on memory functions, hippocampal amyloid deposition, serum cytokine, and BDNF levels in rats with an LPS-induced model of neuroinflammation.

## Materials and Methods

***Ethical statement***

All experimental procedures were conducted in accordance with the European Convention for Protection of Vertebrate Animals used for experimental and other scientific purposes. For our research we obtained permission from the Ethics Committee at the Medical University of Plovdiv (protocol № 2/19.04.2018) and the Animal Health and Welfare Directorate of the Bulgarian Food Safety Agency (permit № 4/09.12.2015).

***Drugs and reagents***

PMX, LPS *E. Coli* O55, and Rat BDNF Elisa Kit were purchased from Sigma-Aldrich; Rat IL-10, TNF-α, and TGF β1 Platinum ELISA kits (eBioscience) were used. 

***Animals***

In this study, we used male Wistar rats (200 ± 20 gr body weight). They were housed and maintained under standard laboratory conditions (12 hr:12 hr light-dark cycle, 22 ± 2 °C temperature, 55 ± 5% humidity) with free access to food and water. All experiments were carried out during the light part of the day.

***Experimental design***

The rats were randomly divided into 5 groups (n=8) as follows:

1^st^ group: control group: saline 0.1 ml/100 g bw,

2^nd^ group: LPS 250 mcg/kg bw, 

3^rd^ group: PMX 0.5 mg/kg bw + LPS 250 mcg/kg bw, 

4^th^ group: PMX 1 mg/kg bw + LPS 250 mcg/kg bw, 

5^th^ group: PMX 3 mg/kg bw + LPS 250 mcg/kg bw.

PMX was given via oral lavage 1 week before LPS administration and throughout the whole experiment. The rats received an intraperitoneal injection of LPS in 5 consecutive days (days 8 to 12). Blood for the immunological assay was collected on day 20 (Table 1). The samples were obtained following the Guidelines for Blood Sample Collection in Small Laboratory Animals.

***Behavioral tests***

*Novel object recognition test (NORT)*

NORT is commonly used to assess exploratory behavior and recognition memory in rodents (17). The experiment was carried out in two consecutive days in an open Plexiglas box (60 cm long, 60 cm wide, 40 cm high). The experimental protocol included 3 phases: habituation, exploration (investigation), and testing. All rats were left to settle in the test box for 5 min in the presence of no objects. During the exploration phase, the rats were allowed to explore two identical objects for 5 min. The testing phase was performed 24 hr later. One of the items used in the exploration session was replaced with a novel one and the rats were allowed to investigate them for 5 min. The time during which the animal explored the novel and the familiar object was detected. We used the following formula for calculation of the Recognition index (RI) 

$\mathrm{EI}=\frac{N}{N+F}\times 100$

N –time for exploring the novel object, F-time for exploring the familiar 

*Y-maze*

Y-maze and spontaneous alternations are widely used to assess spatial working memory in rodents and are based on rodents’ natural exploratory instincts (18). It is constructed of black Plexiglas and consists of three arms interconnected at 120 °. The arms were equal (50 cm long, 10 cm wide, and 30 cm high) and randomly labeled А, В and С. The spontaneous alternation tests were conducted on two consecutive days: one day of training and a retention test on the next day. The animal was placed in the middle of the setup and allowed to investigate the other arms for 5 min. An alternation is consecutive entry into the three different arms of the maze. For example ABC, BCA, CAB, CBA, etc. Spontaneous alternation (SA) was determined by: 

$\mathrm{SA}\left(\%\right)=\frac{\mathrm{Number of alternations}}{\mathrm{Total number of entries}–2}\times 100$

*One way step-through inhibitory “passive” avoidance test*

The step-through passive avoidance apparatus (UgoBasile, Italy) basically consists of two-compartments: one black and one white, brightly illuminated. They are connected by a sliding automatic door. The experiment was run in two consecutive days - for learning and memory assessment, respectively. On the 1^st^ day, each animal was set down in the bright compartment and got access to the dark one following a door delay of 7 sec. When the animal entered into the dark compartment the door slid down and the animal was subjected to a short-lasting aversive stimulus (electrical foot shock for 9 sec, intensity 0.4 mA). The time (in seconds) spent in the illuminated compartment was recorded. The memory retention test was performed 24 hr later (on the 2nd day). We used the same experimental setup but no shock was delivered to the animal (19). The maximum stay in the illuminated compartment was 178 sec (cut-off time).

*Loco-motor activity (activity cage) test*

An automatic device (activity cage, UgoBasile, Italy) was used for evaluation of spontaneous horizontal and vertical movements. The apparatus consisted of an electronic unit and an infra-red beam cage with two sensors - for horizontal and vertical movements. The activity was registered for 5 min. 

*Samples collection*

We used pyrogen and endotoxin-free collecting tubes. Samples were centrifuged (for 10 min) following careful clotting and serum removal. The latter was aliquoted and frozen at -70 °C.

*Immunological assay*

BDNF, IL-10, TNF-α, and TGF-β1 levels were detected in rats’ serum taken on day 20 of the experiments. Solid-phase ELISA was used following the manufacturer’s directions. Absorbance reading was performed at 450 nm and a standard curve was used for calculating serum concentration. The observed parameters were not detected below the following levels: TGF-1β: 8 pg/ml; IL-10: 1.5 pg/ml; and TNF-α: 11 pg/ml. The variation of intra and inter-assay reproducibility was as follows: for BDNF 12 pg/ml; IL-10 1.5 pg/ml; for TNF-alfa 11 pg/ml; and for TGF-beta 1.8 pg/ml.

*Tissue collection*

The rats were decapitated following deep thiopental-induced anesthesia (30 mg/kg bw). Brains were scooped and chilled in iced water (20). The cerebellum and ¼ of the rostral frontal lobes were removed with a surgical blade. The two hemispheres were completely separated through the interhemispheric fissure. Superior and inferior colliculi of the midbrain were located; the surrounding brain tissues were pulled away and the hippocampus was revealed. The latter was separated from the rest of the brain and extracted.

*Histological assay*

10% buffered formalin solution was used for hippocampus fixation. This was followed by tissues dehydration with graded series of alcohol and with subsequent paraffin embedding. 6 µm thick sections were cut by a microtome. In the next stage, the sections containing the hippocampus were dewaxed, rinsed, and stained with Congo-Red (20 min; Diapath, Italy). An optical light microscope (Olympus ВХ51) was used to capture the amyloid plaques in the hippocampal regions. The presence of amyloid deposition was qualitatively verified by the intensity of brown-reddish staining (21).

***Statistical analysis***

IBM SPSS Statistics 19.0 was used. Data are presented as mean±SEM. Analysis was performed with one-way ANOVA, followed by Bonferroni and Games–Howell *post hoc* test depending on the homogeneity of variance. Statistical significance was considered at *P*<0.05.

## Results

***Effects of PMX on learning and memory in rats with LPS-induced model of neuroinflammation***

*NORT*

**Figure 1:**
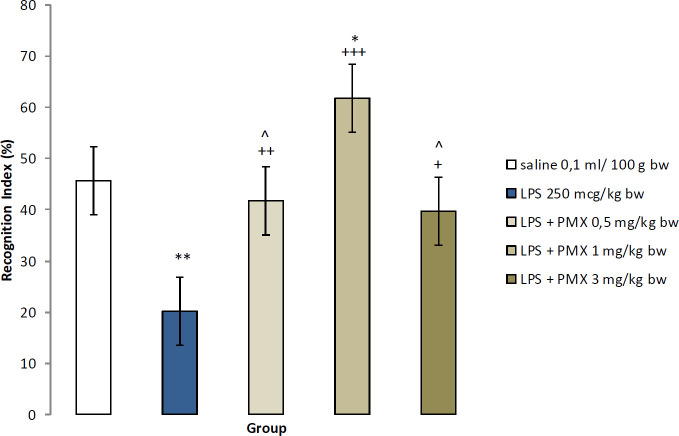
Effects of PMX on RI in rats with LPS-induced model of neuroinflammation. Data are expressed as mean±SEM (n=8).^ *^P<0.05 compared with saline; ^**^P<0.01 compared with saline; ^+^*P*<0.05 compared with LPS;^ ++^*P*<0.01 compared with LPS;^ +++^*P*<0.001 compared with LPS; ^^^*P*<0.05 compared with LPS+PMX 1 mg/kg bw PMX: pramipexole; LPS: lipopolysaccharide

RI was significantly decreased in LPS-treated rats in comparison with saline (*P*<0.01). The rats treated with PMX at a dose of 1 mg/kg bw had increased RI compared with saline (*P*<0.05) and the LPS-challenged rats (*P*<0.001). The other two experimental groups with PMX (0.5 and 3 mg/kg bw) had increased time in exploring the novel object compared with LPS-challenged animals (*P*<0.01 and *P*<0.05, respectively). In rats treated with PMX 1 mg/kg bw, significant increase of the RI was observed compared with the other two experimental groups (*P*<0.05) (Figure 1).

*Y-maze*

**Figure 2:**
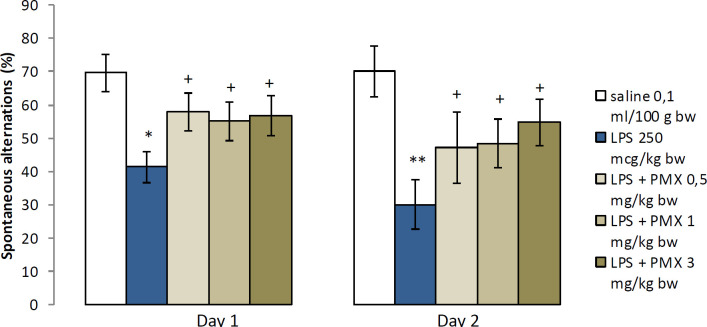
Effects of PMX on SA in rats with LPS-induced model of neuroinflammation. Data are expressed as mean±SEM (n=8). *P<0.05 compared with saline; **P<0.01 compared with saline; +*P*<0.05 compared with LPS

In the Y-maze task, LPS-treated rats showed a statistically significant reduction in the percentage of SA in comparison with saline during training (*P*<0.05) and memory retention test (*P*<0.01). We did not observe a significant difference in spontaneous alternations between the three experimental groups treated with PMX and the vehicle control group. However, the animals treated with all doses of PMX significantly increased the percentage of alternation when compared with the LPS-treated rats during the two experimental days (*P*<0.05) (Figure 2).

*One way step-through inhibitory “passive” avoidance test*

**Figure 3:**
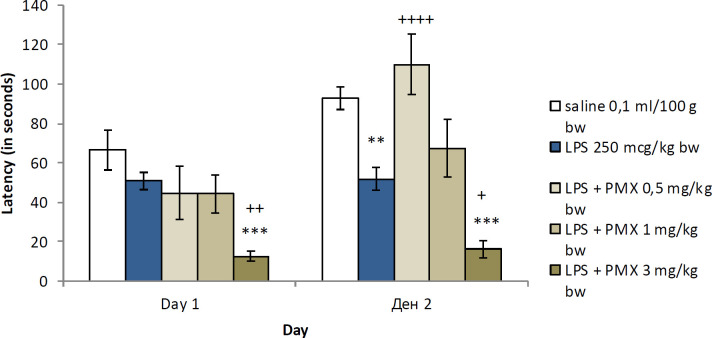
Effects of PMX on step-through test in rats with LPS-induced model of neuroinflammation. Data are expressed as mean±SEM (n=8). **P<0.01 compared with saline; ***P<0.001 compared with saline; +*P*<0.05 compared with LPS; +*P*<0.01 compared with LPS

^++++^ *P*<0.0001 compared with LPS

PMX: pramipexole; LPS: lipopolysaccharide

The LPS-treated group significantly decreased the reaction time on the 2^nd^ day (*Р*<0.01) compared with the vehicle group. The comparison between the three groups with LPS and PMX and the saline control showed that the animals with LPS and 3 mg/kg bw PMX had decreased latency during the two days of testing (*Р*<0.001). When compared with the animals from the LPS group the rats with LPS and the lowest dose of PMX (0.5 mg/kg bw) had significantly increased latency time during the memory retention test (*Р*<0.0001), whereas the animals with the highest dose of PMX (3 mg/kg bw) had decreased latency during the two experimental days (*Р*<0.01 and *Р*<0.05 respectively) (Figure 3). The highest dose of PMX significantly decreased latency compared with the lowest dose of PMX (*P*<0.05 on the 1^st^ day and *P*<0.01 on the 2^nd^ day) and the medium dose of PMX (*P*<0.05 on both experimental days). During the memory retention test PMX at dose of 0.5 mg/kg bw significantly increased the time for entering the dark compartment compared with the other two experimental groups (*P*<0.05 and *P*<0.01, respectively).

*Activity cage test for locomotor activity*

**Figure 4:**
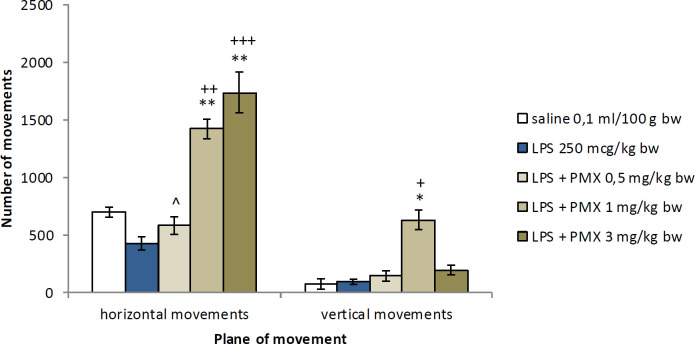
Effects of PMX on locomotor activity in rats with LPS-induced model of neuroinflammation. Data are expressed as mean±SEM (n=8). ^*^P<0.05 compared with saline; ^**^P<0.01 compared with saline; ^+^*P*<0.05 compared with LPS; ^+^*P*<0.01 compared with LPS

^+++^ *P*<0.001 compared with LPS; ^^^P<0.05 compared with LPS+PMX 1 and 3 mg/kg bw

PMX: pramipexole; LPS: lipopolysaccharide

LPS-challenged rats did not show a significant decrease in the number of movements when compared with the saline-treated animals. The group with LPS and PMX at a dose of 1 mg/kg bw had significantly increased number of horizontal and vertical movements in comparison with the vehicle group (*P*<0.01 and *P*<0.05 respectively) as well as the LPS-treated group (*P*<0.01 and *P*<0.05, respectively). The animals injected with the highest dose of PMX (3 mg/kg bw) had significantly increased horizontal movements compared with both controls (*P*<0.01 and *P*<0.001, respectively). The lowest dose of PMX had significantly decreased number of horizontal movements in comparison with the other two experimental groups (*P*<0.05) (Figure 4).

***Effects of PMX on IL-10, TNF-α, TGF-β1, and BDNF serum levels in rats with LPS-induced model of neuroinflammation***

*BDNF serum levels*

**Figure 5:**
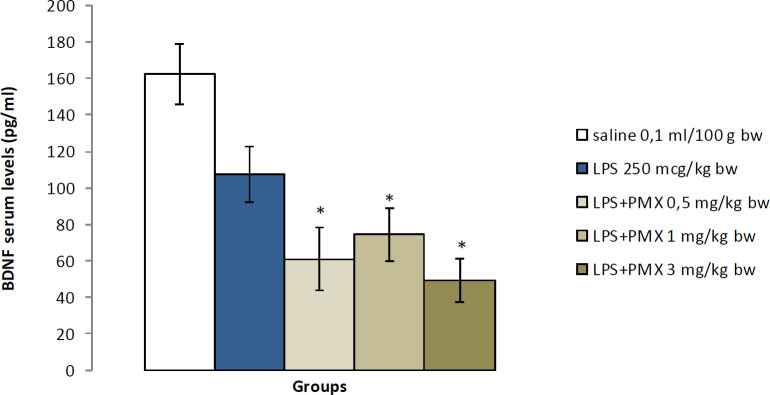
Effects of PMX on BDNF serum levels following multiple LPS administration. Data are expressed as mean±SEM (n=8). ^*^P<0.05 compared with saline

PMX: pramipexole; LPS: lipopolysaccharide; BDNF: brain-derived neurotrophic factor

The LPS-treated group had insignificantly reduced BDNF levels compared with saline. All groups with LPS and PMX at doses of 0.5, 1, and 3 mg/kg bw had significantly decreased BDNF serum concentration when compared with the vehicle control group (*P*<0.05) (Figure 5).

*IL-10 serum levels*

**Figure 6.**
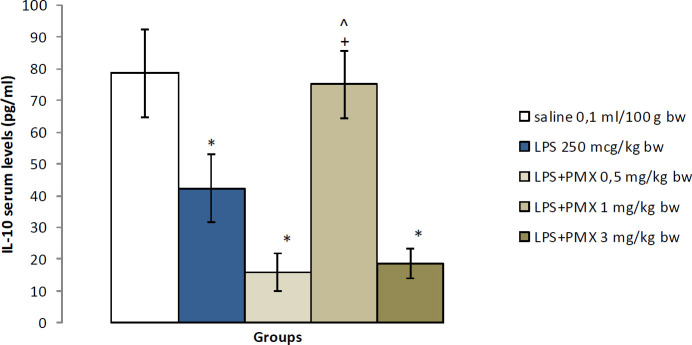
Effects of PMX on IL-10 serum levels following multiple LPS administration. Data are expressed as mean±SEM (n=8). ^*^P<0.05 compared with saline; ^+^*P*<0.05 compared with LPS

^^^ *P*<0.05 compared with LPS+PMX 0.5 and 3 mg/kg bw

PMX: pramipexole; LPS: lipopolysaccharide

The animals from the LPS group showed significantly lower serum levels of IL-10 when compared with saline (*P*<0.05). The group with LPS and PMX at a dose of 1 mg/kg bw had significantly increased serum concentration of IL-10 in comparison with the rats which received only LPS (*P*<0.05). The rats treated with PMX at doses of 0.5 and 3 mg/kg bw had notably reduced IL-10 serum levels compared with the saline-injected animals (*P*<0.05). A statistically significant increase in IL-10 was observed when comparing PMX 1 mg/kg bw with the other two doses of PMX (*P*<0.05) (Figure 6).

*TNF-alfa and TGF-beta1 serum levels*

Serum levels of TNF-α and TGF-β1 remained under the detectable minimum in all experimental groups following multiple LPS administrations.

***Effects of PMX on amyloid deposition in rats with LPS-induced model of neuroinflammation***

**Figure 7:**
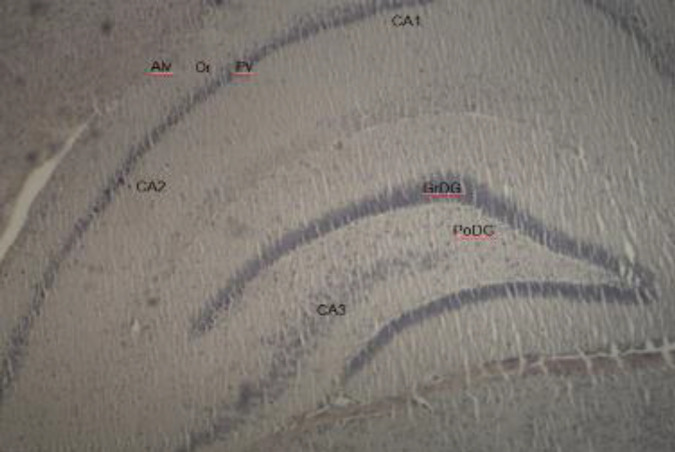
Photomicrograph of hippocampal tissue of naïve rats. CA1 (cornu ammonium 1), CA2, and CA3 zones, granule cell layer of dentate gyrus (GrDG) and polymorphic cell layer of dentate gyrus (PoDG); alv-alveus; Or- stratum oriens; Py- stratum pyramidale. х 100

**Figure 8:**
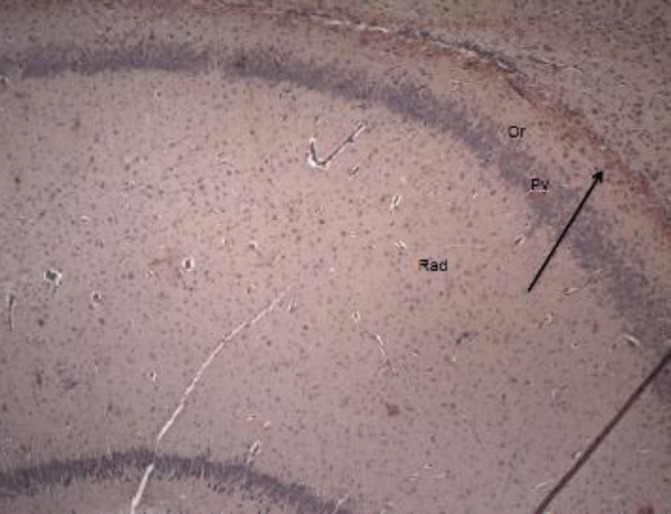
Photomicrograph of hippocampal tissue of LPS-treated rats – high-intensity brown-reddish staining in stratum oriens (amyloid deposition). Or- stratum oriens; Py- stratum pyramidale; Rad- stratum radiatum х 100

**Figure 9:**
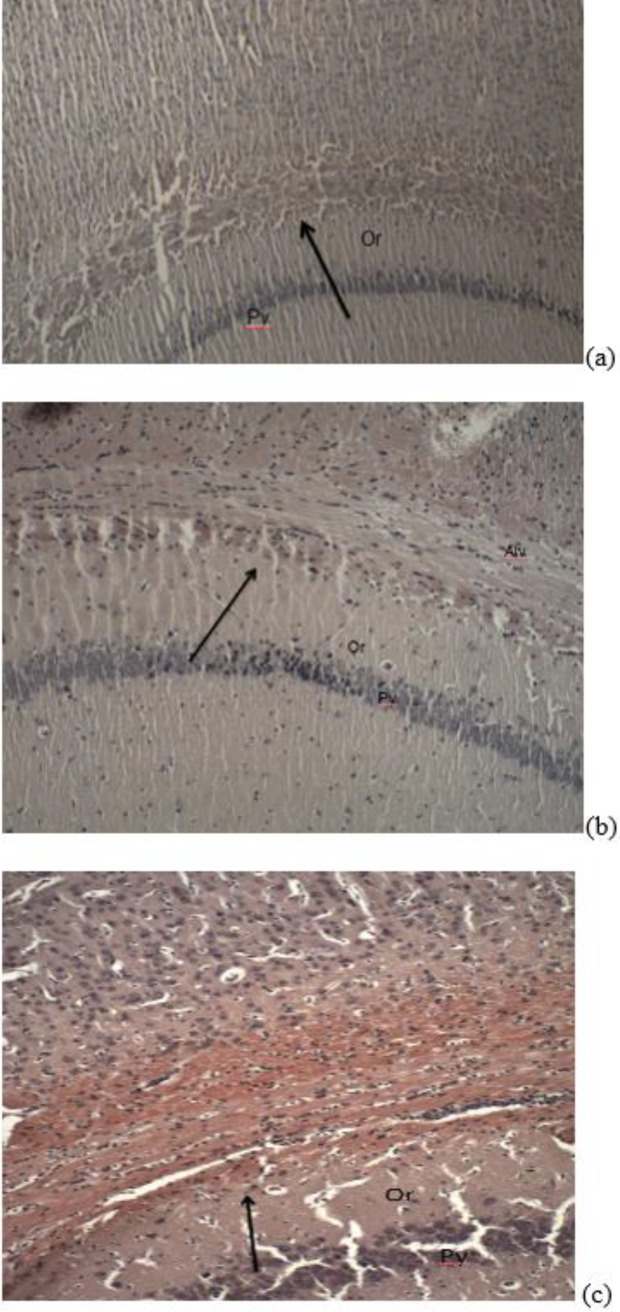
(a). Photomicrograph of hippocampal tissue of LPS and 0.5 mg/kg bw PMX treated rats – low-intensity brown-reddish staining. alv-alveus; Or- stratum oriens; Py- stratum pyramidale х 200; (b) Photomicrograph of hippocampal tissue of LPS and 1 mg/kg bw PMX treated rats – low-intensity brown-reddish staining. alv-alveus; Or- stratum oriens; Py- stratum pyramidale х 200; (c) Photomicrograph of hippocampal tissue of LPS and 3 mg/kg bw PMX treated rats - high-intensity brown-reddish staining. Alv- alveus; Or- stratum oriens; Py- stratum pyramidale х 200

Amyloid deposition in hippocampal structures was determined by using Congo red staining. It did not show abnormalities in the cytoarchitectonic of the hippocampus in naïve saline-treated rats (Figure 7). Histological examination revealed that multiple LPS administration in rats led to amyloid deposition in stratum oriens of the hippocampus of LPS-compromised rats (Figure 8). Administration of PMX (0.5 and 1 mg/kg bw) significantly reduced the amyloid deposition in hippocampal tissues, whereas the highest dose of PMX did not show a marked difference with the LPS-challenged rats (Figures 9A–C).

## Discussion

In our study, we investigated the impact of PMX a dopamine receptor agonist on learning and memory processes in animals with LPS- model of neuroinflammation. The results showed that PMX improved memory in three different behavioral tests dependent on different brain structures. We could assume that the memory improving effect of PMX is mediated by hippocampal and cortical structures.

NORT is widely used for investigation of recognition memory in rodents. The main brain structure involved in this task is the hippocampus. Broadbent *et al*. established that hippocampal lesions cause anterograde memory impairment assessed by the NORT (22). Animal studies suggest that the dorsal hippocampus might be essential for the formation of NORT memory (23). In our study PMX improved long-term recognition memory assessed 24 hr after the exploration session. The main mechanism involved in memory consolidation in the hippocampus is the long-term potentiation (LTP) (24). Dopamine has an essential role in normal hippocampal functioning and LTP (25). We may speculate that PMX exerts its memory ameliorating effect by stimulating dopamine receptors. 

Step-through passive avoidance is one of the most widely used experimental tests for pharmacological studying of learning and memory. Memory assessment is based on the increased latency before the animal steps through the gate between the dark and light chambers. Generally, one trial is enough to evaluate long-term memory. The behavioral response is dependent on the hippocampus but parahippocampal structures may also be involved (26). In the current study PMX at a dose of 0.5 mg/kg bw improved memory consolidation in comparison with the LPS-challenged rats, probably due to stimulation of dopaminergic transmission. Additionally, the neuroprotective properties of this drug might be related to the observed effect since we found that the lowest dose of PMX decreased amyloid deposition in the hippocampus. Surprisingly, the highest dose of PMX worsened memory processes compared with both controls. In the locomotor-activity test, the same dose showed a significant increase in horizontal movements compared with saline and LPS-group. The increased motor activity and the shortened latency for entering the dark chamber might be considered false-negative results. It could be related to the increased dopamine levels in CNS which is a psychomotor stimulant and could provoke anxiety-like behavior. Despite that, histological examination revealed that PMX at a dose of 3 mg/kg bw did not reduce the amyloid presence in hippocampal tissues. 

The rats treated with LPS and PMX had increased spontaneous alternations in the Y-maze. The latter is used as a behavioral trial for assessing hippocampal integrity. Experimental data showed that in naïve rats the rate of SA was approximately 65–70% of total arm entries and in rodents with hippocampal lesions this rate dropped beneath this level (27). The LPS-treated rats had decreased percentage of SA below 45% which could indicate the presence of hippocampal injury in our model of neuroinflammation. During the memory session, PMX at a dose of 3 mg/kg bw had increased alternations above 50% but did not reach the values registered in the saline-treated rats. Dopaminergic stimulation in the hippocampus is the probable mechanism by which PMX restores memory in LPS-compromised rats. Spontaneous alternations are also used as a measure of spatial working memory (28). It is dependent on neuronal encoding in the prefrontal cortex (29). Thus, the performance in the Y-maze task might be reliant on the prefrontal cortex along with the hippocampus. This could explain the difference in the results between NORT and the Y-maze. 

Significantly lower IL-10 levels were observed in LPS-treated rats compared with saline. Experimental data suggest that bacterial LPS may cause or enhance dopaminergic neurodegeneration in rats and mice (30). In mixed cultures of neurons and glia, dopaminergic neurons were found to be more sensitive to the neurotoxic effect of LPS as compared with non-dopaminergic neurons (31). Dopamine plays an immune-regulatory role (32). The decreased IL-10 level in rats with LPS-induced model of neuro-inflammation is probably due to dopamine deficiency. These data could explain the low IL-10 levels in animals with LPS-induced neuroinflammation. 

PMX at a dose of 1 mg/kg bw significantly increased IL-10 levels in LPS-challenged rats. Dopamine has immuno-modulatory functions and regulates cytokine secretion, e.g., it stimulates IL-10 release by LPS-stimulated mice splenocytes (32). Dopamine receptors are expressed by human T-cells, B cells, monocytes, eosinophils, and neutrophils as well as by murine T- and NK cells (33). We could assume that PMX increases IL-10 production by stimulating D_2_- and D_3_- receptors located on immune cells.

In NORT and Y-maze, PMX improved recognition and spatial memory in LPS-compromised rats. As aforementioned these tasks are partly dependent on the hippocampal integrity. IL-10 is important for the maintenance of some aspects of the hippocampal functions. A study found that in IL-10 knock-out mice LPS administration leads to difficulties in hippocampus-dependent learning and spatial memory task (34). Based on our results we may speculate that PMX enhances recognition and spatial memory by increasing IL-10 levels. 

Systemic administration of LPS in experimental animals activates microglial cells with subsequent release of pro-inflammatory cytokines, (21). *Qin* *et al.* found that intraperitoneal LPS injection stimulates hepatic synthesis of TNF-α and increases its serum concentration, which in turn increases its brain production. They concluded that serum TNF-α levels are high in the first few hours following LPS-injection and gradually decrease to basic levels within the 9^th^ hour, whereas its levels in the brain remain high for approximately 10 months (35). These could explain why TNF-α levels were under the detectable threshold in the LPS-treated group. Blood samples were collected on the 20th day of the experiments when TNF-α serum levels are expected to be reduced to their basal levels (below the detectable minimum) despite the persisting brain inflammation. 

LPS-challenged rats showed reduced serum BDNF levels compared with the saline control. Several *in vivo* studies examine the effect of inflammation on BDNF expression. LPS reduces BDNF mRNA levels in the rat hippocampus (36) and some cortical regions (37) after intraperitoneal administration. A study found that LPS reduces BDNF in the prefrontal cortex and hippocampus but increases its levels in the nucleus accumbens (38). There is evidence that BDNF mRNA levels are significantly reduced in the rat hippocampus at the 4th hour after intraperitoneal LPS injection (36). One limitation of our study is that we evaluated BDNF serum levels not its hippocampal concentration . Klein *et al*. found that concentration of BDNF in whole blood correlates with its hippocampal levels in rats, suggesting that peripheral BDNF measurement could be used as a biomarker for its hippocampal concentration (39). Based on these data, we may assume that our results about BDNF serum levels correlate with those in the hippocampus and therefore multiple intraperitoneal LPS administrations decrease hippocampal BDNF levels.

Existing data for the effect of PMX on BDNF levels are controversial. In primary cell cultures of mesencephalic dopamine neurons, PMX increases BDNF synthesis through D_3 _receptor stimulation (40). Researchers found that PMX reduces BDNF mRNA levels in some brain structures (nucleus accumbens and amygdala) and increases them in others (ventral tegmentum). PMX probably has a different effect on BDNF expression in different brain structures – an increase of this neurotrophic factor in some structures leads to a compensatory decrease in others (41). As aforementioned BDNF serum levels correlate with its hippocampal concentrations in rats. Our results show indirectly that in a rat model of neuroinflammation PMX does not increase hippocampal BDNF expression. 

Chronic intraventricular infusion of LPS in rats results in increased β-amyloid precursor mRNA in the hippocampus (42). Lipopolysaccharide increases β- and γ-secretase activity, possibly by stimulating the production of inflammatory cytokines (43). In the present study histological examination of hippocampal structures from LPS-compromised rats showed accumulation of amyloid plaques in the stratum oriens of the hippocampus. PMX suppressed their deposition, probably due to its anti-inflammatory effect. One of the possible mechanisms by which PMX exerts this effect is by increasing production of the anti-inflammatory cytokine IL-10. This in turn suppresses the activity of the pro-inflammatory factors responsible for amyloid formation. 

**Table 1:** Experimental design of the study - duration of treatment and days of conducting memory tasks

Day	1	2	3	4	5	6	7	8	9	10	11	12	13	14	15	16	17	18	19	20
РМХ	+	+	+	+	+	+	+	+	+	+	+	+	+	+	+	+	+	+	+	+
LPS								+	+	+	+	+								
Step-through test													+	+						
NORT															+	+				
Y-maze																		+	+	
Sample collection																				+

PMX; pramipexole; LPS: lipopolysaccharide

## Conclusion

PMX administration in rats with LPS-induced neuro-inflammation improved hippocampal-dependent memory and exerted immuno-modulatory effects by increasing the production of the anti-inflammatory IL-10. Our data also revealed that this dopaminergic agonist reduced amyloid plaques in the hippocampus. The current study gives new insights on the therapeutic usefulness of PMX in the treatment of cognitive decline that accompanies neurodegenerative disorders.
